# Value and Limitations of Broad Brush Surveys Used in Community-Randomized Trials in Southern Africa

**DOI:** 10.1177/1049732318809940

**Published:** 2018-12-17

**Authors:** Virginia Bond, Fredrick Ngwenya, Emma Murray, Nothando Ngwenya, Lario Viljoen, Dumile Gumede, Chiti Bwalya, Jabulile Mantantana, Graeme Hoddinott, Peter J. Dodd, Helen Ayles, Musonda Simwinga, Sandra Wallman, Janet Seeley

**Affiliations:** 1 Zambart, School of Public Health, University of Zambia, Lusaka, Zambia; 2 Department of Global Health and Development, Faculty of Public Health and Policy, London School of Hygiene & Tropical Medicine, London, United Kingdom; 3 Desmond Tutu TB Centre, Stellenbosch University, Tygerberg, Cape Town, South Africa; 4 Africa Health Research Institute, KwaZulu-Natal, South Africa; 5 School of Health and Related Research, University of Sheffield, Sheffield, United Kingdom; 6 Department of Anthropology, University College London, London, United Kingdom

**Keywords:** Broad Brush Survey, BBS, Zambia, South Africa, community-randomized trials, qualitative

## Abstract

We describe and reflect on a rapid qualitative survey approach called “Broad Brush Survey” (BBS) used in six community-randomized trials (CRTs)/studies in Zambia and South Africa (2004–2018) to document, compare, classify, and communicate community features systematically for public health and multidisciplinary research ends. BBS is based on a set sequence of participatory qualitative methods and fieldwork carried out prior to a CRT intervention and/or research by social scientists to generate rapid community profiles using four key indicators: physical features, social organization, networks, and community narratives. Profiling makes apparent similarities and differences, enabling comparison across communities and can be facilitated by an ideal model of open-closed systems. Findings have provided practical outputs (e.g., community profiles) and academic opportunities (e.g., community typologies). The BBS approach enables complex social landscapes to be incorporated in CRTs. This method has proven to be useful, adaptable and to have multidisciplinary appeal.

## Introduction

In 2004, 24 communities (16 in Zambia, eight in South Africa) were designated to be part of a community-randomized trial (CRT) to reduce tuberculosis (TB) incidence (Zambian and South African TB and AIDS Reduction Study—ZAMSTAR; Ayles et al., 2013). As the lead social scientist in the trial, I (first author) vividly remember staring at the spread of communities across six provinces in Zambia and across the Cape Metropole and winelands in Western Cape, South Africa, wondering how we could adequately and meaningfully represent the social context of TB and HIV in these scattered and diverse communities to the wider multidisciplinary research team. As an applied anthropologist, I was used to understanding and communicating the complexity of a multilayer context, but I was far less familiar with the scale of this undertaking.

There is often a challenge, when commencing a new trial or study, to describe systematically community features that might matter and why, and to feed this into planning and design. Pearce and Merletti (2006), exploring the application of complexity theory (“the study of complex adaptive systems” p. 515) note, “there are very few examples of the use of the complexity theory in epidemiology, but there are many examples of epidemiological problems for which the complexity theory is relevant.” In this article, we describe a “Broad Brush Survey” (BBS) qualitative approach that I and others have turned to when conducting social research to orient CRT implementers to the dynamics of the study communities in which they are working. We describe the evolution, theory, specifics, and application of BBS as a method within, or related to, six CRTs. We reflect on whether the BBS approach has made context and variability more explicit and relevant, either potentially or in practice, at different stages of the CRTs, that is, has BBS managed to communicate and make complexity count? Our primary aim in this article is to establish the values and limitations of BBS, first, as a rapid pretrial qualitative approach that is compatible with epidemiology and second, as a method which can provide data on complex urban communities both within and across different communities. In addition, we define core premises upon which BBS is based. As Pearce and Merletti (2006) argue, “‘Local’ research that is grounded in a particular population is more likely to produce findings that address universal themes and issues than is research that attempts to strip away the population context” (p. 518).

## The Evolution of BBS

The term “Broad Brush Survey” and original emphasis is derived from Valdo Pons (1969, 1993a, 1993b, 1996), a sociologist, who aimed to capture a broad overview of a place by sketching the surface and mapping the broad parameters as a starting point for the research to follow. Valdo Pons was adamant that this sketch impression was arrived at by walking around, sketching, and observing and not asking any questions. It was very much an overview, a broad but comprehensive sweep. In 1993, Wallman worked with Valdo Pons on a research study of women’s social problems and options for health care in a densely populated area in Kampala, Uganda’s capital city (Wallman, 1996). BBS was the first step in this research. Subsequent adaptations of BBS to public health problems were to follow, including those in this article. Valdo Pons, original BBS has thus been superseded and BBS is now about what is going on “on the ground.”

The BBS has become a social research approach for collecting, collating, and comparing data about communities that can be useful for different reasons to CRTs, community, and social research enquiry. To qualify as BBS, social research needs to run ahead of CRT intervention or research and to inform both. BBS needs to retain its landscape scope and a set of core methods in sequence, and it needs to compare communities by systematically and rapidly observing key features of communities. These key features are contained in the meta-indicators of physical features, social organization, networks, and community narratives, developed by Wallman, Bond, Montouri, and Vidali (2011) out of earlier work on the diversity of urban systems (Wallman, 2003) and the later application of BBS to CRTs. See Table 1 for a summary of the development and adaptation of community feature indicators. The comparison of communities can be aided by using an ideal model of open-closed urban systems (Wallman, 2003) that elucidates the relative diversity and interrelatedness of the meta-indicators of community features.

**Table 1.** Developing Indicators of Diversity for Urban Systems.

Initial 10 Indicators→ 2003	Adaptation 1 Indicators→ 2005	Adaptation 2 Indicators→ 2005	Meta-Indicators 2011
London, Turin, Kampala (Wallman, 2003)	Zambia, South Africa (ZAMSTAR, Wallman, 2011)	Zambia, South Africa (ZAMSTAR, Wallman, 2011)	Zambia, South Africa (Developed by Wallman, 2011, Applied in BHOMA & HPTN 071 [PopART]Bond et al., 2016 )
Industrial structure Industrial type Employment opportunities Travel to work Travel facilities Labor movement Housing options Gatekeepers Criteria for membership Political membership	Livelihood Mobility Topography Housing options Interest groups Community identity Interactions TB popular Knowledge & perceptions TB treatment patterns Special features	Livelihood Mobility Topography Housing options Interest groups Interactions	Infrastructure and population (physical, countable, features) Social organization (relation of people to place, choice among the options) Networks (relations of people to people, patterns of inclusion & exclusion, control of local resources) Narratives (stories about us, who is in the moral community, who belongs here)

*Note.* ZAMSTAR = Zambia South Africa TB and HIV Reduction; TB = tuberculosis; BHOMA = Better Health Outcome through Mentoring and Assessment; HPTN 071 [PopArt] = HIV Prevention Trial Network Population Effects of Anti-Removal Treatment to reduce HIV Transmission.

### BBS and the Open-Closed Model of Urban Systems

Following Wallman’s Ugandan research in 1996 and building on an interest in the comparison of urban systems, Wallman later combined data from studies she had led on localized urban systems in two London boroughs (1982, 1984), the Ugandan research (1996), and Turin, Italy (2003) to develop an ideal-type model on open-closed urban systems (2003). The ideal model was abstracted from 10 diverse indicators (see Table 1) of urban systems.

Building on a principle established by Jacobs (1961), that diversity is vital for the viability of urban systems, Wallman argues based on comparing these indicators and urban systems that this

succession of field studies in different cities and parts of cities has indicated a systematic logic which broadly accounts for these better or worse outcomes. As local systems, some areas are relatively more open and more heterogeneous than others. These are routinely more adaptable in the face of change or incursion, with more fluid, more “open” inter-cultural communication. (Wallman, 2003, p. 1)

Hence, each indicator can be described according to either being “open” (heterogeneous and open to change/influence/outsiders) or “closed” (homogeneous and resistant to change/influence/outsiders). The housing options indicator, for example, can be classified as “open” if the urban system has many different types of houses or “closed” if the urban system has housing of identical types.

**Figure 1.**
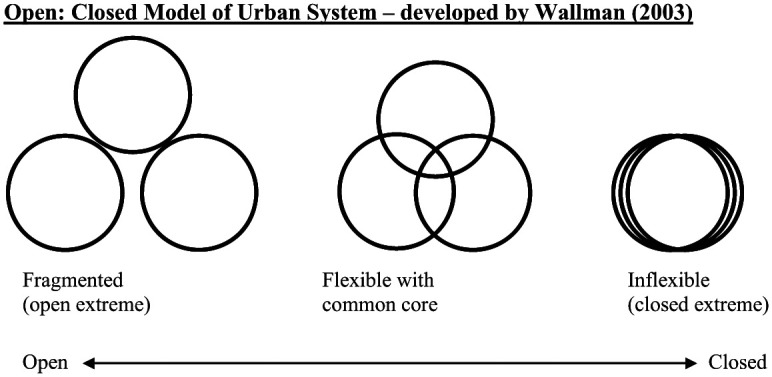
Open-Closed model of urban systems. Source. Wallman (2003).

Both Jacobs (1961) and Wallman (2003) further point out that each indicator is related to the other indicator within the same urban system and this “interrelatedness” (Wallman et al., 2011, p. 17) cannot be enumerated. To understand these relations within the ideal model, Wallman uses the concepts of “boundary systems” and the “network effect” to look at how these indicators are interrelated in a local system and to subsequently get a sense of the whole urban system (Wallman, 2003, pp. 9–10). Comparing one local system with another helps assess the degree of open “ness” and close “ness,” ranking them in comparison with each other according to whether they are “more” or “less” open/closed (2003, p. 18). This is illustrated in Figure 1 (developed from Wallman, 2003, 2011), where each ring represents housing, work, and social life. When these local resource domains overlap tightly (e.g., who you work with is who you live with and socialize with), the likelihood of interaction and communication with the wider outside, and of adapting to change, are more limited. On the other extreme is a chaotic openness, where the “connected at the core” (what Wallman, 2011, refers to as a “strong localist identity,” p. 24) is lost. The most resilient system in times of “drastic change” (Wallman, 2011) is the flexible, open but with a common core type because it is a system open to intervention/outsiders/new ideas, connected at core and able to draw on connections outside the core if necessary (Wallman 2003, pp. 24–25).

Initially ZAMSTAR, which provided an unusual opportunity to include social context in restricted randomization (see Bond, 2011; Murray, 2010; Sismanidis et al., 2008), and the then other main CRTs included in this article used BBS data to draw on this open-closed urban systems model in different ways, having initially organized their data around key features of communities, captured in Table 1. Table 2 summarizes the use of the open-closed model across the CRTs and studies.

**Table 2.** Use of Open-Closed Model Across CRTs and Studies.

CRT/Ancillary Study	Use of the Open-Closed Model
ZAMSTAR	Randomisation: Each community placed along open-closed continuum and classified as open (heterogeneous) or closed (homogeneous) relative to each other. This binary typology was incorporated as a “co-variate” in restricted randomisation process to spread interventions across different types of communities.
CODA	Retrospective mixed method analysis: Aware of ZAMSTAR typology of open-closed but focused more on key locations relevant to social mixing between adults, and adults and children, and TB transmission. Current retrospective analysis might use open-closed to help understand the role of social and spatial engagement in TB transmission.
BHOMA	Practical: Each community placed on an open–closed continuum and implications for uptake of intervention reflected on by study team.
HPTN 071 (PopART)	Applied and mixed method analysis: Each community classified as either more open or closed, and one pithy sentence included on this in short narrative summary report. Considering using this to draw up a typology to feed into current analysis of HIV prevalence baseline data to see if it helps explain variation in HIV prevalence, including some striking outliers. BBS data will also be used to help explain anticipated variation in primary outcome (HIV incidence) across communities.
P-ART-Y	Applied: Intervention planning discussions drew on open-closed community classification and stakeholder mapping
Society in Transition	Not used: However, based on BBS data, developed a model of social change which focuses on similar dimensions that rise out of using meta-indicators and open-closed model with a stronger focus on social justice. Their model focuses on comparing availability of options, equity of access to resources and social networks/cohesion and how these link to perceived control (see Ngwenya et al., 2018, p. 79).

*Note.* CRT = community-randomized trial; ZAMSTAR = Zambia South Africa TB and HIV Reduction; CODA = Contact Observations of Daily Activities; TB = tuberculosis; BHOMA = Better Health Outcome through Mentoring and Assessment; BBS = Broad Brush Survey; P-ART-Y = PopART for Youth.

## The Six CRTs

The series of CRTs, which form the basis of this analysis can be viewed as de facto case-studies for evaluating the BBS approach. Nearly all were designed to address an urgent public health issue in Southern Africa from 2004 to 2018 (Ayles et al., 2013; Ayles, Sismanidis, Beyers, Hayes, & Godfrey-Faussett, 2008; Dodd et al., 2015; Hayes et al., 2014; Shanaube et al., 2017; Stringer et al., 2013) and managed in the field by the same institutions and with the same core multidisciplinary team. One other study in South Africa, led by another institution, used the BBS approach as a lead into a CRT and other studies, providing valuable insights on the application of BBS beyond the earlier Southern African teams.

The BBS approach was used as the first social scientific activity (and often the first trial activity) in all six of the aforementioned studies. Out of these six examples of the BBS approach, four were part of a larger qualitative component and the CRT design. The four CRTs evaluated interventions to reduce TB and/or HIV and/or mortality, with the implementation period ranging between 1 and 4 years, in many communities (21–42, urban and rural) with total population sizes of 450,000 to 1.2 million (Ayles et al., 2013; Ayles et al., 2008; Dodd et al., 2015; Hayes et al., 2014; Shanaube et al., 2017; Stringer et al., 2013). The two other studies used the BBS approach explicitly linked to a CRT: one is an ancillary study nested within a CRT, which aimed to investigate an aspect of the wider trial, namely social contacts and TB (Dodd et al., 2015); another is a qualitative study in South Africa, in four KwaZulu-Natal communities, which was conducted prior to a CRT of HIV transmission and two intervention studies (Ngwenya et al., 2018). Table 3 gives an overview of each CRT or study, including the design, primary outcome or aim, interventions (if any) and key references, and explains the purpose and timing of the BBS, and the broader qualitative design.

**Table 3.** Community-Randomized Trials (and Ancillary Studies) and “Their” BBS in Zambia and South Africa, 2004–2018.

CRT or Ancillary Study	Primary Outcome/Aim & Intervention/s (if Any)	Period	Study Country & Communities	BBS: Purpose and Timing	Broader Qualitative Design
CRT: ZAMSTAR study. Trial registration no: ISRCTN36729271 Design: 2 × 2 factorial design, 4 Arms (including one control Arm). Key references: Ayles et al. (2013); Ayles, Sismanidis, Beyers, Hayes, and Godfrey-Faussett (2008), Sismandis et al. (2008).	Aim: Reduction of tuberculosis prevalence after 3 years of interventions. Interventions: Enhanced case finding of TB cases ± Household Counseling of Index TB patients	2004–2011	Countries: Zambia and South Africa Communities: 24 (16 Zambian across 5 provinces, 8 South African in Western Cape. Mainly urban, 4/24 rural) Population size: approx. 1 million	Purpose: To gain an understanding of social interaction, the local economy, the mobility of individuals, daily activities, perceptions of TB, TB treatment options, TB stigma and patients’ experiences of health services within each community. Timing: 2005. Prior to intervention. First research activity.	BBS + more intensive qualitative fieldwork during the intervention (observations of enhanced case finding, household counseling and in-depth interviews with TB patients) + ethnography of converging impact of TB, HIV, and food insecurity
Ancillary study: CODA, a social contact study relevant to TB nested within and performed at the end of ZAMSTAR. Design: Combining social contact, TB incidence and TB prevalence data. Key reference: Dodd et al. (2015).	Aim: To identify patterns of social contacts that correlated with TB transmission, including social mixing patterns among children and adults. Social contact data derived from cross-sectional face-to-face survey of randomly selected adults (*n* = 3,528).	January–March 2011	As above (same countries and communities as ZAMSTAR)	Purpose: (a) to update a previous BBS in these communities; (b) to provide insight into likely patterns of behavior and especially settings of adults and child social mixing to feed into the design of the CODA questionnaire. Timing: 2011. Post ZAMSTAR intervention. Last research activity. Note: Carried out in 16 Zambian communities only.	BBS + pilot of social contact quantitative questionnaire.
CRT: BHOMA. Trial registration no: NCT01942278 Design: Stepped-wedged design. Key reference: Stringer et al. (2013).	Aim: Reduction of mortality in those aged <60 years, measured through three annual household surveys. Interventions: clinical mentoring, data monitoring, community engagement.	2011–2014	Country: Zambia. Communities: 42 (one province, three districts, mainly rural with 3/42 urban) Population size: approx. 450,000	Purpose: To map out a wide understanding of the local systems in 8/42 communities (remote rural, rural on main road, district town center) and how they affected the uptake of government health care services. Timing: 2011, 2014. Pre and post BHOMA intervention.	BBS (baseline & endline) + pilot of verbal autopsy questionnaire.
CRT: HIV Prevention Trial Network (HPTN) 071 (PopART)—registration no: NCT01900977. Design: Cluster-randomized, 3 Arms (including one control arm). Key reference: Hayes et al. (2014).	Aim: Reduction of HIV incidence at population level after 3 years of interventions. Intervention: HIV combination prevention package delivered at household level & including the annual offer of HIV testing and immediate initiation of Antiretroviral Therapy (ART) in one Arm.	2012–2018	Countries: Zambia and South Africa. Communities: 21 (12 Zambian across 4 provinces, 9 South African in Western Cape, all urban) Population size: approx. 1 million.	Purpose: (a) Rapidly gauge key community features and community perceptions and experiences with HIV prevention, treatment and care options; (b) Identify catalysts and barriers to the uptake of different aspects of the intervention; (c) Inform and interpret the trial findings and act as baseline for further social science enquiry. Timing: 2012–2013. Prior to intervention. Carried out alongside rapid census.	BBS + “Story of the Trial” documentation of intervention implementation & post intervention activities + qualitative cohort of adults with different HIV decision pathways (e.g., tested/not tested, HIV-positive/HIV negative, on ART/not on ART).
CRT Ancillary study: P-ART-Y. Trial registration no: NCT01900977 Design: As above for HPTN 071 (PopART) + cross-sectional survey in Arm C communities of young adults (*n* = 2,120). Key reference: Shanaube et al. (2017).	Aim: Increased self-reported uptake of HIV counseling and testing among young people aged 15–24 over the previous 12 months. Intervention: Youth-optimized PopART interventions. For example, youth counselors.	2015–2017	As above (same countries and communities as HPTN 071 PopART)	Purpose: To map and observe adolescent gathering places and activities in the clinic and community to inform intervention. Timing: October–November 2015. Prior to intervention. Note: BBS 2012–2013 data also analyzed for observations on young people.	BBS 2013 (above) + BBS 2015 + adolescent HIV stakeholder survey + qualitative cohort of adolescents with different HIV decision pathways (e.g., tested/not tested, HIV-positive/HIV negative, on ART/not on ART).
Study: Society in Transition—what is the impact on health? Data used in subsequent CRT on reducing HIV transmission in women & mortality in men. Key reference: Ngwenya et al. (2018).	Aim: Experiences and perceptions of transmission, treatment and prevention of HIV and TB. Note: Initial study provided data and/or BBS approach for 3 subsequent studies including 1 CRT.	2015–2016	Country: South Africa Communities: 4 (one district in KwaZulu Natal, 2 peri-urban, 2 rural). Population size: approx. 90 000	Purpose: Rapid qualitative assessment of (a) perceptions of local boundaries; (b) gathering places; (c) definitions of HIV “hotspots” and TB ‘hotspots’; (d) community perceptions of drivers of the HIV epidemic, and the occurrence of TB. Timing: Carried out alongside epidemiological and mapping research of HIV “hotspots” & “cold spots.”	BBS only.

*Note.* BBS = Broad Brush Survey; CRT = community-randomized trial; ZAMSTAR = Zambia South Africa TB and HIV Reduction; TB = tuberculosis; CODA = Contact Observations of Daily Activities; BHOMA = Better Health Outcome through Mentoring and Assessment; P-ART-Y = PopART for Youth.

## The BBS as a Method

The BBS used rapid, qualitative and participatory methods to systematically carry out observational activities in health, economic, and social settings within a geographically bounded place, and, to interview representative groups and individuals in that place to gather qualitative data around a key research question directly related (in this instance) to the relevant CRTs on public health. This systematic social research was usually carried out in several places (and countries), lending itself to “broad-brush” comparative, rapid, and applied analysis on key features and interactions between people and place (and people and people within that place) around a core research question (Bond, 2011; Wallman et al., 2011). For example, in the ZAMSTAR trial, BBS was carried out in all 24 communities in Zambia and South Africa in 2004–2005, and the aim was to understand domains of TB in each community by rapidly gathering data. Certain participatory methods were used to elicit the different domains (see Figure 1). For example, the use of space by age, gender, and occupation (“who hangs out where and for how long”) and places where people congregate (and any links made between this and TB transmission by local residents) used maps of TB “hotspots” generated through a discussion with a local health committee, a transect walk which observed the “hotspots” and structured observation in gendered spaces (e.g., a water point for women). Observations on local options for livelihood; the range of local housing; class, ethnic, unique, leadership, local economy characteristics; and range of social interactions were captured through the transect walk and structured observation at markets, entry/exit points, and transport hubs, and over weekends and at night. A snapshot of use of the local health center was documented through a structured observation. The range of and opinions about TB treatment options, local etiology of TB, and level and type of TB-related stigma was collected through a historical timeline of TB with elders and in-depth interviews with TB patients and TB specialists (Bond, 2011; Sismanidis et al., 2008; Wallman et al., 2011). Collectively, these areas of enquiry provide preliminary indications of features of difference in the local context that could shape the uptake of TB services and interventions. The other studies similarly developed key research questions and matched research methods to these.

The aim, key questions, and sequence of research activities, which includes details on personnel, material, data software and logistical requirements, time span, sites, tools, process and outcomes, were drawn up as a Standard Operating Procedure (SOP) for BBS fieldwork (see Appendix in Wallman et al., 2011), accompanied by research tools and informed consent forms and adapted for each CRT or related study.

BBS fieldwork in each community was carried out in a block of time over a period of 5 to 15 days. Fieldwork was often staggered but, depending on the size of the team, often concurrent in more than one community. Having some short break in-between communities allowed for a period of de-briefing, data management, and writing up. Usually, all communities, which were part of the CRT, were included, but sometimes resources limited BBS to a smaller, representative number of communities. Hence, in BHOMA, eight out of 42 communities were selected representing three different districts and deep rural, rural main road, and district center in each. In Society in Transition, four communities were selected from a wider study demographic and health surveillance area covering 90,000 people, to represent high/low prevalence, and peri-urban/rural (Ngwenya et al., 2018).

The fieldwork was carried out by a social scientist, often assisted by a research assistant (usually a local resident) to work in a pair. The research assistant was either a local resident recruited as a guide for the fieldwork period (usually through local health committees) or a trained social science research assistant located in (and a resident of) the community. Research assistants ranged in educational background from school leavers to degree holders. An effort was made to have a man and woman pair of researchers in each community to facilitate insights on men and women, and sometimes also to provide additional security (see below). Ethnic composition and language competence were also important considerations, for example, in Western Cape, in both ZAMSTAR and HPTN 071 (PopART), it was essential to have Xhosa and Afrikaans speakers as well as to be sensitive to mistrust of outsiders, particularly “white” researchers. Table 4 summaries the BBS social science research teams in each CRT or study.

**Table 4.** BBS Social Science Research Teams.

Ancillary Study	Senior Social Scientists		Field Team Social Scientists		Local Research Assistants	
	Men	Women	Men	Women	Men	Women
ZAMSTAR	0	3	2	5	12	4
CODA	0	1	1	0	16	16
BHOMA	0	1	1	1	8	8
PopART	2	3	2	3	4	6
P-ART-Y	1	1	3	6	2	6
Society in Transition		1	2	2		

*Note.* BBS = Broad Brush Survey; ZAMSTAR = Zambia South Africa TB and HIV Reduction; TB = tuberculosis; CODA = Contact Observations of Daily Activities; BHOMA = Better Health Outcome through Mentoring and Assessment; P-ART-Y = PopART for Youth.

Time spans, resources, and distance to the field dictated team numbers, composition, and organization. Financial costs for BBS have varied from $17,000 to $200,000. Training of research teams for BBS usually took 1 week and could be conducted centrally or at the community level. The training aimed to familiarize fieldworkers with the participatory techniques used in BBS and equip them with qualitative research skills such as facilitating, probing, reflexivity, observing, asking open-ended questions, community entry and exit, and the writing of textual data. Focus group discussion, in-depth interviewing skills, and logistics were also usually included. Researchers were orientated to research tools and practiced using research tools in sessions and teams and by carrying out observations. Training also aimed to give fieldworkers an understanding of the CRT and their role within the CRT (including introducing the study) and to reflect on community entry and ethical issues they may face while doing fieldwork. For HPTN 071 (PopART), all researchers had to additionally take and pass on-line Good Clinical Practice courses. The teams were prepared for circumstances where they may witness or experience a crime (particularly in South Africa), for the event that they would be asked questions about health conditions related to the trial, and for situations where they might be asked for social support or need to refer people to support services. The core teams also underwent training in data management and software packages used for coding data (namely, NVivo or ATLAS.ti Version 7).

The research activities were carried out in a sequence that started with broader observations and narrowed down to more structured observations in gathering places, entry/exit points, and other spaces and times of relevance to the question (e.g., local health facilities and other treatment options and at night and during the weekend). The observation periods, which stretched over 3 to 5 days, formed the essential sequence and set of activities for BBS because they were the activities that captured the meta-indicators (see Figure 1, essential tools). Any focus group discussions and individual interviews took place after the observation period and began to narrow the research down to focus on the health issue at the core of the respective CRT. There was no perfect order to the sequence, but rather an approach to move from wider to narrower observations, allowing the narrower to build on the wider observations and allows for more opportunistic possibilities.

**Figure 2.**
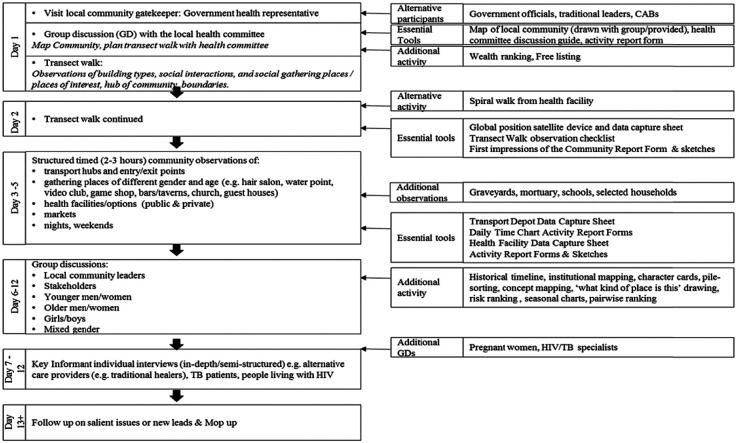
BBS set of methods in sequence.

Figure 2 reflects the flow of research activities and both essential tools and additional tools that have been used. An opening research activity with health gatekeepers (.g., in Zambia this would be a statutory neighborhood health committee) directly informed the subsequent activities (e.g., which places should be observed, who should be interviewed). Common participatory techniques used include focus group discussions, social mapping, transect walks, free-listing, observation checklists, daily time charts, and historical timelines. Hand-held Global Positioning Systems (GPS) were also frequently used for the transect walk, forming a “bread crumb trail” (Murray et al., 2009). Additional participatory techniques included spiral walks, concept-mapping, character cards, wealth-ranking, drawing and statements on cards, pairwise ranking, seasonal calendars, and exit interviews.

Participants were selected on the basis of being representative of either gender and age (older men, younger men, older women, younger women) or their particular expertise and perspective on the public health issues (health committee member, members of nongovernmental organizations, church leaders, health care workers including community lay volunteers, traditional healers, people living with the health condition). Recruitment was based on membership of a group, referral by the health committee, health facility staff and influential community leaders, age and gender networks, geographic spread and representativeness, and being “on the spot.”

In addition to the pre-trial BBS, a “top-up” BBS was carried out at a later stage in two CRTs, to carry out observations of a particular research group and/or question related to a new ancillary study. In CODA, additional observations of interactions between children and adults in locations defined as “casual contact locations” (e.g., churches and bars; Dodd et al., 2015, p. 157) and households were carried out prior to a quantitative survey. For P-ART-Y, additional observations of young people in gathering places and in the community were carried out prior to an intervention (Shanaube et al., 2017). In both studies, the earlier BBS in the same communities was first reviewed for appropriate data, and the “top-up” BBS were focused and carried out over a shorter period.

The total number of structured observations in different locations and participants across all the communities for each CRT are summarized in supplemental table. Participants were counted only if they gave written consent and engaged in a more formal research activity (e.g., a group discussion) and not if an informal conversation was held during an observation. As reflected in the table, BBS enables observations and interactions (including discussions and interviews) with a large range of locations, people, and communities in a relatively short period of time.

The analysis of BBS data was carried out in three distinct phases. The first phase consisted of rapid analysis, carried out through routine debriefing of researchers either during fieldwork or at end of community fieldwork, an analysis workshop with social science team/s carried out immediately after data collection was completed and by writing up rapid analysis outputs. Respective researchers took up responsibility for “their” set of communities (where they carried out the fieldwork). These outputs, summaries of each communities in different forms (e.g., short/long narratives, matrices, community flyers), technical reports (again both brief and more detailed), and community typologies were disseminated to the trial team, district and national stakeholders, communities, and funders usually within 4 to 6 months of completing fieldwork. Table 5 summarizes these applied outputs across the CRTs and studies. The outputs were most commonly used to provide communities with profiles they could discuss and use in a broad range of ways (e.g., when communities were seeking funding for development projects), to provide trial implementers with practically useful information to tailor the implementation of the intervention by study context (e.g., identifying issues of relevance for community engagement, research, and intervention) and to inform epidemiological structured questionnaire design (e.g., see Dodd et al., 2015; Hargreaves et al., 2016). Often BBS data helped community engagement teams determine how the study should be introduced and provided them with data to develop a community message. Less usual and accomplished in ZAMSTAR was to draw on a community typology based on rapidly synthesizing BBS data and using the open-closed model of urban systems (Wallman, 2003; Wallman et al., 2011) to constrain randomization, thus randomly allocating different interventions across different types of communities (see Bond, 2011; Sismanidis et al., 2008).

**Table 5.** BBS Rapid Analysis Applied Outputs.

CRT/Study	General	Community Specific
ZAMSTAR	Introducing ZAMSTAR text (for study, with community engagement) Developing messaging for the CRT BBS results, Open-closed continuum (for restricted randomization, to Principal Investigators & statisticians) Presentations to wider teams, funders	Item: Community feedback flyers. 3 pages Format: Cartoon visuals. Language: Non-academic language. Presented in large font, word, photocopied, not dense. Structure: What is BBS? What do people do every day and where do they go? Community mobilization and leadership. What do people say about TB? TB treatment options? Is there TB stigma? What was the point of this work? What next?
CODA	Questionnaire design (social contacts)	None
BHOMA	Questionnaire design (verbal autopsy) Presentations to wider teams, funders	Item: Community brochures. 2 pages. Format: Cartoon visuals. Language: Report language. Presented in color, publisher, dense. Structure: What is BBS? What words were used for serious illnesses? Perceptions about serious illnesses (ranking, causes, stigma), treatment options, health initiatives, community support structures, reasons for going to the health center, relationships between health providers and users, delivery options for pregnant women, experiences of death.
HPTN 07 (PopART)	Introducing PopART text (for study, with community engagement) Developing messaging for the CRT Technical Report to Funder (detailed + short) Presentations to wider teams, funders	Item: Short narrative summary report. 6 pages. Language: Report language. Word, crisp. Photocopied. Structure: Purpose and methodology, orientation to the community, the people in the community, list of stakeholders, implications of perceptions and experiences relevant to HIV and HIV services, other contextual factors relevant to social experiences of health, summary of implications for PopART (research, intervention, community engagement), emerging themes, research participant and activity details Item: Matrix summary report. 11 pages. Format: Matrix layout, with findings and implications side by side. Language: Report language. Word, dense. Photocopied.Structure: What kind of place is this? (4 meta-indicators—physical features, social organization, networks, narratives). Local stakeholders. Perceptions and experiences of HIV. HIV stigma. Emerging themes for this place. Participant and activity detail. Item: Long community narrative. 20 pages. Structure: Same structure as short summary report but with more detail. Not shared as widely.
P-ART-Y	Technical report to funder (a component) Presentations to wider teams, funders (including team asked to determine interventions who used narrative reports for discussion and design in a workshop)	Item: Community adolescent narrative. 7 pages. Format: Map of community included. Mixed BBS findings with trial census data, including stakeholder survey findings. Language: Report language. Word, dense. Photocopied. Structure: orientation to community, features of significance, adolescent gathering places, experiences of adolescents and HIV prevention, experiences of adolescents and HIV testing, experiences of adolescents living with HIV, one page summary of findings, stakeholder matrix, map.
Society in Transition	Presentations to wider teams, funders	Flyers

*Note.* BBS = Broad Brush Survey; CRT = community-randomized trial; ZAMSTAR = Zambia South Africa TB and HIV Reduction; TB = tuberculosis; CODA = Contact Observations of Daily Activities; BHOMA = Better Health Outcome through Mentoring and Assessment; P-ART-Y = PopART for Youth.

The second BBS analysis phase was more manual, with operational analyses focused around a particular theme, often during the intervention/research period and in response to trial issues and conference opportunities. For example, in HPTN 071 (PopART) BBS data were analyzed and built upon to explore challenges with linking people living with HIV to HIV services. A subsequent short report on findings was shared with the wider trial team and helped adjust the intervention process. This phase could also lead to further social science enquiry. For example, identifying a pattern that requires further research such as understanding stigma related to “being seen” at the local health facility (see Bond, Nomsenge, et al., 2016). The third phase was driven by academic outputs and is preceded by transcription, coding, and finer analysis, with qualitative data analysis programs often helping the managing of the data (NVivo or ATLAS.ti). Throughout the duration of the trials (and beyond), BBS was a platform and baseline for other qualitative and/or epidemiological analyses carried out during the CRT. These either used a mixed method approach (see Bond, Chiti, et al., 2016; Murray et al., 2013) or used BBS data alone (see Bond, Hoddinott, et al., 2016; Murray et al., 2013; Ngwenya et al., 2018; Viljoen et al., 2016). These analyses of BBS data drew on one community (e.g., Murray et al., 2009), one country (Murray et al., 2013), a selection of communities (see Bond, Chiti, et al., 2016), or included all the communities (e.g., Ngwenya et al., 2018) and countries (e.g., Bond, Hoddinott, et al., 2016).

## Ethics

The ethical approvals are detailed in Table 6. BBS was usually approved as part of the main trial research, unless it was separately funded or conducted independent of a specific trial. Governmental health authority clearance was always also obtained in South Africa and Zambia, and for all the CRTs and studies, Community Advisory Boards were already existing or set up.

**Table 6.** Ethical Clearances.

CRT/Ancillary Study	Ethics Clearance for BBS
ZAMSTAR	University of Zambia, Stellenbosch University, and the London School of Hygiene and Tropical Medicine ethics committees approved the study in 2004, including BBS. Additional approval obtained for Murray’s master’s analysis of BBS in 2007 from Stellenbosch University ethics committee (Murray, 2010).
CODA	University of Stellenbosch Health Research Ethics Committee (N04/10/173), the University of Zambia Biomedical Research Ethics Committee (007-10-04), and the London School of Hygiene and Tropical Medicine Ethics Committee (A211 3008) approved the study in 2010, including BBS.
BHOMA	University of Alabama at Birmingham, the University of North Carolina, the University of London School of Hygiene and Tropical Medicine, and the University of Zambia Research Ethics Committees approved the study in 2011, including BBS (004-12-08).
HPTN 071 (PopART)	University of Zambia Humanities and Social Sciences Research Ethics Committee (011-11-12), Stellenbosch University Health Research Ethics Committee (N12/09/056), and the London School of Hygiene and Tropical Medicine (6278) ethics committees approved BBS ahead of and independent of the main study in 2012.
P-ART-Y	University of Zambia (011-11-12), Stellenbosch University and the London School of Hygiene, and Tropical Medicine ethics committee approved the study in 2015, including the BBS.
Society in Transition	University of KwaZulu-Natal Biomedical Research Ethics Committee (BE197/15) approved the study in 2015.

*Note.* CRT = community-randomized trial; BBS = Broad Brush Survey; ZAMSTAR = Zambia South Africa TB and HIV Reduction; TB = tuberculosis; CODA = Contact Observations of Daily Activities; BHOMA = Better Health Outcome through Mentoring and Assessment; P-ART-Y = PopART for Youth.

Written informed consent was obtained from all research participants engaged in group discussions, in-depth interviews, and key informant interviews, and for any photographs where individuals could be identified. For observations, verbal consent was obtained from appropriate authorities (e.g., the health staff in charge of the health facility or the proprietor in charge of a bar or salon). Outputs and coded data removed community, place, job title, and person names. Community names were often replaced with codes when findings were disseminated outside of the country or in publications.

A key ethical issue encountered was lack of safety in the field—particularly in South Africa during BBS fieldwork for ZAMSTAR and HPTN 071 (PopART) and at nights and weekends and around the time of the monthly welfare grants pay out. For example, in HPTN 071 (PopART), the South African BBS research team was warned by local residents about security concerns, particularly theft of equipment, violent assault, and the risk of rape in women (Abrahams et al., 2014). Precautions taken included researchers working in mixed gender pairs, matching research ethnicity to the dominant ethnicity and language of any one community, working closely with governmental and local authorities, referral to appropriate services (for residents and researchers when necessary), withdrawal from the field or activities in the face of heightened threats (Abrahams et al., 2014), and supplementing discussions and walking with photographs and driving if necessary.

## Reflections on the Values and Limitations of BBS as a Method

### Value of BBS

BBS draws on various and specific qualitative methods in sequence to produce a qualitative still-life of communal characteristics. It highlights the value of population-based research and/or public health research paying more attention to local characteristics across communities and tailoring research or intervention design based on this contextual detail. Intrinsically it was regarded as useful by all CRTs. The value of BBS lies in the flexibility of the method and an ability to feed directly into community engagement, intervention design, and other research components and to be appreciated across disciplines. It was valued by the research teams for rapidly capturing wider local features that allowed for community comparison and highlighted community capability, and for generating data that allowed for producing applied outputs swiftly, including short community profiles, as well as providing a data set for finer and retrospective analysis.

As a method, the flexibility of BBS to triangulate methods and incorporate different methods and tools is a strength. Reflecting on the disciplines underlying BBS, it is immediately evident that the BBS under scrutiny used methods that are rooted most clearly in rapid participatory research and ethnography, and influenced by sociology, human geography, and politics. In circumstances where BBS was not widely known as an approach, the tendency was to initially refer to it as “formative” and/or “baseline” and/or “rapid qualitative assessment” with a gradual shift to using the term “BBS.” Murray (2010) describes ZAMSTAR as having participatory features because the wider CRT

aimed to establish a more long term enabling presence in research communities by implementing interventions within the framework of local health facilities. The trial employed local people and hopes that successful interventions will be absorbed and sustained by communities and their health services. (Murray, 2010, p. 13)

Differences across countries also showed that flexibility in approach to team organization was important. In South Africa, the relative close proximity of communities to the institution’s office in ZAMSTAR and HPTN 071 (PopART) made both centrally based research assistants and other logistics easier, whereas in Zambia, the distance to some communities limited both the choice of research assistants and time in the field. For all teams, BBS training in participatory methods and ethics, fieldwork experience, data management and analysis as well as the multidisciplinary opportunities were valuable capacity building for early and mid-career African social scientists.

The synergy highlighted by Murray (2010) between community engagement and BBS was experienced by all the social science teams in the CRTs and studies. Simwinga et al. (2016) explicitly linked the HPTN 071 (PopART) BBS with the community engagement principle of first learning about a community from a community and then building on this knowledge in research and intervention delivery. Simwinga et al. (2016) illustrated how BBS data were used to formulate consistency in introducing the CRT including certain disease and CRT terms, to develop intervention messaging and to identify strategies for representation (including alternative health providers). This “systematic consultation” was an “essential first step” to enabling an understanding of the community.

In addition, at the outset of the CRT, BBS data were said to also inform health services, intervention structures, and research components. Across components, BBS was identified as producing data that identified community capability of responding to change, including a public health intervention, according to, for example, the history of the community, population mobility, “target” populations, social cohesion, varying levels of pride and belonging, and functional structures and services. BBS was valued for generating data that allowed comparison across communities. Common features and differences (e.g., housing types) consistently emerged from the approach that lent themselves to comparison along a “greater/lesser extent” continuum.

A significant observation relating to how the analytical boundaries of “the community” are defined by CRTs emerged from BBS findings. CRTs often relied on population catchment areas of a health facility to define a “community,” but these catchment areas did not necessarily constitute “a community,” with heterogeneity within communities emerging from BBS data. For example, ethnic divisions between colored, Afrikaans speakers and black, Xhosa speakers led to two distinct communities within a population catchment of a health facility defined as “a community” in ZAMSTAR and HPTN 071 (PopART). Murray (2010) refers to communities within “a community” as “multiple cityscapes” (p. 55).

BBS was experienced as working with other disciplines who found BBS accessible. The social science teams involved in multicountry CRTs found that African epidemiologists in the team were more open to adopting and understanding BBS, but that other trial epidemiologists and bio-statisticians based outside the region were slower to come on board. The two core institutions involved have observed how BBS led to more respect for social science, with other disciplines involved in the CRT increasingly turning to the teams to ask for data around different research areas and intervention challenges.

BBS was experienced as generating “vast” amounts of data across many communities in a short period of time. For example, in HPTN 071 (PopART), a research pair of graduate researcher and local research assistant collected 25 hours of group discussion recordings and 10 days’ worth of observational field notes per study community in just 2 weeks. Clear data management processes are critical to collate, secure, and access data, and to share data across countries within a multicountry CRT, and this management has improved over time by trial and error for two social science teams involved in many of the CRTs. A key point is ensuring that all team members have a clear and shared understanding of the unifying question that all the data are intended to answer. The rapid analysis carried out through debriefing sessions, summaries, and short structured reports was more able to capture what immediately jumped out and the bare bones of the landscape, including the indicators. This research analysis was less inductive and more about identifying data relevant to the public health problem.

Applied outputs were prolifically, successfully, and consistently produced. Compelling and crisp community specific profiles were rapidly produced in all CRTs and studies (see Table 5). In ZAMSTAR and HPTN 071 (PopART), an effort was made to given copies of the profiles to appropriate people visiting the field and to make soft copies available within the African institutions. The shorter profiles, and particularly those in matrix form with clear recommendations for different aspects of the trial (community engagement, research, intervention), were absorbed and used more widely. For example, one district HIV stakeholder team spent a meeting discussing the findings in a BBS HPTN 071 (PopART) short matrix summary and deciding how to address issues raised in the summary. The shorter narrative profiles proved popular with communities, although community feedback and discussion about these was hard to finance and prioritize in the trials. BHOMA provided an unusual opportunity to use BBS findings presented in brochures to reflect on relationships between health providers and users. ZAMSTAR community flyers used the most accessible English; other flyers tended to use more report technical language.

Retrospective analysis of BBS has proved to be insightful and feasible, and the body of data generated from the same 12 communities in Zambia from BBS in 2004, 2011, 2013, and 2015 provides some unusual longitudinal social research data analysis opportunities.

### Limitations of BBS

The limitations of BBS relate to the degree that BBS is embedded in wider trial structures including interventions. They are also linked to limitations in the participatory spin of BBS, the quality of the data being dependent on the presence of social scientists and adequate social science training, and the management of concurrent fieldwork strategies. The data itself are limited in ability to collect more in-depth and nuanced data and to capture what is happening outside the communities. There is also a risk of misrepresenting community boundaries by aligning with trial definitions of community. The longer rapid outputs have proved to have more limited use and the finer analyses of BBS data are time-consuming and harder to prioritize.

If BBS is not embedded within trial and intervention structures, it was harder to make it useful and achieve interdisciplinary engagement. In addition, if different components of the trial were too autonomous and effective communication channels were not in place, it was hard to feed BBS into wider trial activities. For example, in BHOMA, because the intervention and research were carried out by different organizations, it was harder to feed BBS findings back into intervention planning and design, or to do mixed method analysis.

The importance of BBS fieldwork being conducted by social scientists was also evident when assessing the quality of data collected by research assistants alone or the necessarily limited interpretation of the approach by other disciplines. BBS carried out poorly undermines the rigor and value of the approach well done. Reflections on team composition and numbers revealed that too few social scientists, or social science time, limits the application of BBS both practically and academically. This was the experience of BHOMA. Another study, CODA, trained 32 new research assistants and had them all conducting BBS at the same time in 16 different communities with the remote oversight of one social scientist and one experienced research assistant. This experience demonstrated that so many research assistants and concurrent fieldwork undermined quality and management of data, with data from one community having to be disregarded and needing to supplement data with the earlier BBS data.

As a method, BBS is only participatory up to a point. Murray (2010) assessed ZAMSTAR as falling short of the emancipatory ideals of empowering communities that is embedded in participatory techniques, with more weight on data generation. To make BBS more participatory, more intensive community feedback and subsequent action need to be carried out, such as that demonstrated by Rowa-Dewar et al. who used rapid appraisal as an approach in 10 Scottish communities to achieve meaningful public involvement in cancer care. The BBS teams, for example, felt that theater for development at the end of the BBS would be a valuable addition that would enable immediate feedback and quick analysis of the data with the community (Evans, 2017).

BBS is a baseline or top-up approach, which requires more in-depth research to follow in its wake. Research that followed BBS was regarded as more able to comprehensively research these subtler, more invisible characteristics such as stigma and commitment to place, and to capture longitudinal response to intervention over time. Following BBS with in-depth data collection and analysis fits with a recent framework for including rigorous in-depth qualitative research as part of a larger “rapid cycle,” multisite evaluation approach in public health, which allows for both rapid and applied feedback while retaining the depth of information needed (Skillman et al., 2018). In addition, it was widely recognized by the social scientists that household experiences of health conditions and services needed more in-depth qualitative approaches. Hence, most of the CRTs and studies, with the exception of CODA, used BBS as a platform for further qualitative enquiry.

The risk with BBS was also that it reinforced the trial definition of the community, which does not correspond to how people are experiencing community boundaries. Both ZAMSTAR and HPTN 071 (PopART) dealt with this partly by substituting the term “community” with “site,” “community site,” or “place” in some outputs and making this point explicit to the wider team.

BBS also produced limited insights on employment/livelihoods outside the community. It did not generate enough information about job seeking and places of trade and employment (formal and informal) outside of the CRT community boundary, which often had significance to the public health issue in question because of the impact of social mixing and mobility on disease transmission and strains and the reach of the intervention.

The opportunities for quantifying differences within and between communities could be more capitalized than they have been to date by adjusting the design of tools and/or carrying out the community typology more often that was used in ZAMSTAR (Bond, 2011). For example, Murray and colleagues’ (2013) retrospective analysis of the significance of various gathering places for TB transmission in one South African community reflected that the observation check list would have benefited from incorporating TB transmission variables more comprehensively.

Although the short-applied outputs including community profiles were widely utilized, any longer profiles had less use and were only ever read by social scientists revisiting the data or by keen CRT visitors. No flyers were translated into vernacular languages. It was often hard to mobilize resources and trial support for more intensive community feedback.

Furthermore, finer analysis of BBS has proved time-consuming and harder to prioritize than the rapid outputs. Data coding of all the data to facilitate drawing out themes and finer analysis was experienced as taking up considerable time, often eating into time that could be spent on either further data collection, other research tasks (including reviewing literature and engaging with social theory), or analysis. More selective coded and less fine coding are strategies that have proven more efficient and productive for the Zambian and South African teams involved in many of the multiple-country CRTs and studies. Transcripts are both time-consuming and costly (if outsourced) and a more selective approach to transcribing (e.g., not transcribing interviews that did not go well and rather relying on a summary of the interview) is sometimes adopted. Certain activities (e.g., character cards) are both hard to transcribe and code and need to be managed differently at the finer analysis stage. Academic analyses were varied and overshadowed by further qualitative activities and analyses, financial resources, and by other CRT tasks, including another CRT on the footsteps of the previous one.

## Discussion

The inclusion of BBS in a span of CRTs over the last 14 years both speaks to the openness of certain CRT principal investigators to support the approach, the ability of the research teams to be transdisciplinary, the appeal and value of the approach to the wider trial team and stakeholders, and to its adaptability. It also marks a good time to reflect more critically on what it is and is not, what it can and cannot achieve and the future potential of the approach. While examining the concept of “syndemics,” Singer and Clair (2003, p. 434) argue for a “biosocial reconception of disease” which encompasses a “more holistic approach that emphasizes interrelationships and the influence of context” (p. 434) by appreciating the “synergistic interaction of two or more coexistent diseases” (p. 423). They say this is needed to make us “more alert” (Singer & Clair, 2003, p. 434) to social inequities intrinsic to the burden of disease. BBS responds to this syndemic concept. The Society in Transition study that focused on HIV and TB and used the BBS approach exposed how the lack of control over structural and environmental barriers (e.g., opportunities for men to work in rural communities) were more of a priority to local residents than HIV, while also promoting HIV transmission (Ngwenya et al., 2018, pp. 78–79). Similarly, Murray et al. (2013, p. 410 ff.) used ZAMSTAR BBS data to demonstrate how TB was experienced as “unavoidable” in eight Western Cape townships, with this “lack of control” having a “disempowering effect” that reduced treatment seeking.

The value of a holistic approach lies at the heart of social anthropology and is also propounded by the concept of “complexity” (Hawe, 2015; Pearce & Merletti, 2006), which requires “new approaches” for understanding interactions between “the intervention and the context (or system) into which it is placed” (Hawe, 2015, p. 307). Rather than shy away from complexity, it is important to embrace it because as Wallman (2011) spells out, “the variety of local response is not in doubt” and “unexplained local differences get in the way of managing a predictable outcome. Policy implementation is impeded if not confounded by local diversity” (pp. 9–10). Meadows (2009), a systems theorist, echoes the same sentiment: “the same outside event applied to a different system is likely to produce a different result” (p. 2).

The BBS approach grapples with complexity of local social systems by rapidly and systematically gathering data on features of the system “before the intrusive stage, and so to plan for it realistically and more appropriately” (Wallman et al., 2011, p. 207). It is by using comparison between communities that key indicators can be identified and contrasted with each other. This requires both “sameness and difference” (Wallman et al., 2011, p. 198) and a necessary process of selection and abstraction, producing both wide and shallow data on communities. Pearce and Merletti (2006) suggest that “what appears chaotic and unpredictable at one (usually lower) level may be relatively simple and stable at another (usually higher level)” and point out that “to obtain useful knowledge, one must focus on the appropriate level” (pp. 515–517). With BBS, the research framework necessarily shifts from framing the individual toward framing the collective; the “higher” level is achieved by comparing at the very least visible features (e.g., the meta-indicators), with the possibility of using typologies, such as the open-closed model, to classify communities. Whether the open-closed model is used or not, BBS as an approach, with its focus on landscape and more salient features, lends itself both to comparison and broader social issues that narrower approaches to disease-specific questions might overlook.

Exploring the disciplinary roots of BBS has revealed a reach across social science disciplines and in communicating with other disciplines and stakeholders. This is because it has proved “friendly enough” and helpful across a range of people, places, disciplines, and issues (Wallman et al., 2011, p. 12). S. Wallman (personal communication, March 13, 2018) wondered if BBS in its current form is almost “non-disciplinary” and reminds us pragmatically that the point is not to “measure it to a classical ideal” but rather to use it in an applied arena. Historically rooted in sociology, adapted by social anthropology, with tendrils in human geography, development studies, and political ecology, it is now used more widely across the social sciences and can be pushed theoretically to link to complexity, urban systems, and typology. The use of participatory methods, both specific and others added in that make sense to the team and/or question at hand, makes it familiar to social scientists and development experts and makes it adaptable and flexible. The risk is that BBS becomes too loose and too cheap and starts to look like a poor man’s ethnography. Some ways to avoid this wobbliness is to be clearer and more systematic about BBS and to maintain rigor by having social scientists carry out the approach and by pushing the links with theory.

The collaboration between BBS and other core CRT disciplines has included what Béhague, Gonçalves, and Victora (2008) identified as being embedded in epidemiological practice, for example, adapting the CODA questionnaire to local contexts. Reflecting on collaborations between anthropologists and epidemiologists, they comment that collaborating around “real analytical needs” (Béhague et al., 2008, p. 2) is unusual, with collaboration typically being more slow, superficial, lacking in theoretical grounding, and often reduced to the incorporation of oversimplified methods “toolkit” (Béhague et al., 2008). In BBS, it has been easier to demonstrate the usefulness for application and harder to foster a genuine collaboration around analysis, particularly toward the end of CRTs and around the flurry of the primary outcome where numerical findings are at the fore. As Trostle and Sommerfeld (1996) state about the relationship between medical anthropology and epidemiology, “collaboration through merging involves deeper and more equal transfers between disciplines, combining method and theory” (p. 258).

**Figure 3.**
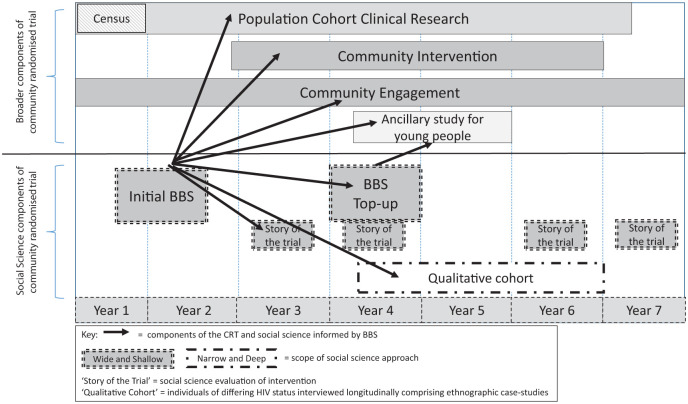
Incorporation of BBS within HPTN 071 (PopART). *Note.* BBS = Broad Brush Survey.

What can BBS achieve on this front? Figure 3 shows the incorporation of BBS within the broader design of the HPTN 071 (PopART) study, highlighting the function of BBS. The qualitative component began with a rapid BBS across all the study communities, providing a “wide and shallow” understanding of social topography. This understanding helped to inform wider research and trial components about the context of the study or trial, and also suggested further social science research topics and questions, similar to the approach advocated by Skillman et al. (2018). The next step in the qualitative component comprised more longitudinal qualitative enquiries about community response to the intervention and more in-depth ethnographic research. The latter provided a more “narrow and deep” understanding of the social reality of HIV. The ethnographic research comprised case studies of small numbers of individuals in a more limited number of communities, pulling out to wider enquiry at the end of trial. Exchange and interaction between qualitative researchers and other more biomedical teams within the CRT were the most intense during community entry and when community response to either intervention or research raises challenges for implementation or when major policy shifts occurred. Importantly, as collaboration and the application of the BBS method to the disease context evolves, several key premises emerge as significant to retaining the integrity of BBS.

## Underlying Premises of BBS

First, BBS is an approach that involves coming out of our disciplinary corners. While rooted in social research, it requires having a shared interest with other disciplines (in the CRT research team in this instance) in a public health issue and an openness to being eclectic. As Meadows (2009) states, “Interdisciplinary communication works only if there is a real problem to be solved, and if the representatives from the various disciplines are more committed to solving the problem than to being academically correct” (p. 183). BBS requires us to be at the very least multidisciplinary in endeavor, and at the most transdisciplinary at heart. Bennett (1995) defines transdisciplinary as “working together to form a common vision not blinkered by differing disciplinary approaches. The common vision can be just an ability to work alongside each other as different disciplines or a trans-disciplinary commitment could ‘push the [disciplinary] boundaries’” (p. 1590) between disciplines (Béhague et al., 2008, p. 1701) to create new, shared conceptual frameworks (Porta, 2014). Respect and trust between disciplines is implicit.

Second, we need to stand back from the granular detail to take in the wider landscape and it is this that BBS brings into view. Wallman (2011, pp. 13–15) uses two metaphors to convey this—a garden and a fish tank. The gardening metaphor draws on the 18th landscape gardening of Lancelot (“Capability”) Brown in the 18th century who focused on “capabilities” in each garden. Both garden and fish tank draw attention to boundaries, perceptible features, and the whole system, and consider what lies within that is clearly visible. This infers the value of initial impressions and intuition (or hunches) and of holistic and socioecological perspectives. The fish tank metaphor draws our focus to not only the place but the people in the place: “What kinds of fish live in it [the fish tank]? What options does it offer them? . . . How do particular fish move in it? Which of the options on offer does each sort of individual take up?” (Wallman et al., 2011, p. 13). BBS hence combines topography and social organization, including population movement, at a moment in time.

Third is that every community is uniquely put together while sharing some organizational and structural features with other communities, and that these shared and different sociological characteristics matter to health. While this may be startlingly obvious to social researchers, we need to remember this is less obvious to disciplines that are more reductionist and positivist. Leaning on complexity theory, Pearce and Merletti (2006, p. 516) argue that epidemiologists need to pay much more attention to the “history, culture and socio-economic structures” of “each population,” they remind epidemiologists that neither people nor populations are “just random collections” and that “Complex adaptive systems have a ‘life’ that is more than the sum of their component parts” (p. 517). They illustrate this by pointing out, for example, that “Risk factors for disease do not operate in isolation but occur in a particular population context” (Pearce & Merletti, 2006, p. 517).

If we accept the premise of particularity, difference, and similarity, we move to the fourth premise, which is that contrast and comparison are key to explain the diversity of local systems (Wallman et al., 2011, p. 12). “The local system is a function of relations between people and place, of the options of topography and infrastructure available to ‘locals’—and of the way they chose among them” (Wallman et al., 2011, p. 12). This comparison of the “framework of possibility” (Wallman et al., 2011, p. 13) relies on two types of uses for qualitative data; data as starting points for developing definition for categorical classification (Porta, 2014, p. 233), for example, more/less, present/absent, and to qualify (describe) the nuances of particular systems/experiences. Comparing and contrasting communities across both registers of qualitative data enables researchers to not only identify differences and similarities but to develop theoretical models that support the typology (Wallman et al., 2011, p. 207). Once qualitative data have been used to classify a community (e.g., more/less mobile), then there are more opportunities for iteration with other forms of CRT statistical data.

The fifth and final premise is that BBS is not intending to predict community response. BBS does not intend to be predictive, but it does intend to be locally sensitive by identifying key features that we are able to systematically “see” and “feel” and that matter to health and health intervention uptake. It is also, however, a “snapshot” be it a “locally nuanced snapshot” (Murray et al., 2009, p. 772). Thus, although BBS conveys features, it is rooted in conveying the importance of structures, connectiveness, options, and equity, and argues that social heterogeneity and social cohesiveness are needed to “receive” public health interventions (Murray, 2010, p. 64; Wallman, 2003), community features uniquely differ in degree and mix and are “systems in process” (Wallman et al., 2011, p. 13) with “change in one element altering the capability of the whole” (Wallman, 2011, p. 16, citing in Jacobs, 1961, p. 433) and leading to a “new outcome and different options.” As Hawe (2015) simply states, “Complexity increases the unpredictability of effects” (p. 207).

## Conclusion

The use of BBS as an approach within CRTs, because of the focus on the landscape and more salient features, lends itself both to comparison and engagement with broader social issues that a narrower focus on disease specific questions might overlook. Disciplinary boundaries are crossed by BBS and can be pushed, and BBS can push theoretical boundaries, but the onus to date has been more on practical outcomes for public health ends. The analysis for this article has allowed key premises to emerge, which underscore how the innovation of BBS in relation to CRTs has proven adaptability, speed, multidisciplinary appeal, and communicative ability to bring the collective features of a community into view. It fits into a larger body of rapid appraisal approaches that demonstrate that “rapid” does not mean “rushed” (Beebe, 2014) and that “rapid can be systematic and rigorous as well as applied" (Rowa-Dewar et al.; Skillman et al.) and adds to this literature the value of comparison and models of urban systems. It reminds us of the value of “watching” the local system, the history, and the people who live there (Meadows, 2009). Moreover, it responds to Jane Jacobs who prefaces her seminal book with the request, “please look at real cities. While you are looking, you might as well also listen, linger and think about what you see” (Jacobs, 1961, p. 1).

## Supplemental Material

BBSinCRTS_Table5__Resubmission_Supplementary_20180803 – Supplemental material for Value and Limitations of Broad Brush Surveys Used in Community-Randomized Trials in Southern Africa Supplemental material, BBSinCRTS_Table5__Resubmission_Supplementary_20180803 for Value and Limitations of Broad Brush Surveys Used in Community-Randomized Trials in Southern Africa by Virginia Bond, Fredrick Ngwenya, Emma Murray, Nothando Ngwenya, Lario Viljoen, Dumile Gumede, Chiti Bwalya, Jabulile Mantantana, Graeme Hoddinott, Peter J. Dodd, Helen Ayles, Musonda Simwinga, Sandra Wallman and Janet Seeley in Qualitative Health Research

QHR809940_Supplemental_Material_REV4 – Supplemental material for Value and Limitations of Broad Brush Surveys Used in Community-Randomized Trials in Southern Africa Supplemental material, QHR809940_Supplemental_Material_REV4 for Value and Limitations of Broad Brush Surveys Used in Community-Randomized Trials in Southern Africa by Virginia Bond, Fredrick Ngwenya, Emma Murray, Nothando Ngwenya, Lario Viljoen, Dumile Gumede, Chiti Bwalya, Jabulile Mantantana, Graeme Hoddinott, Peter J. Dodd, Helen Ayles, Musonda Simwinga, Sandra Wallman and Janet Seeley in Qualitative Health Research
