# Supplementary material for: Value and Limitations of Broad Brush Surveys Used in Community-Randomized Trials in Southern Africa
Source: Qual Health Res. 2018 Dec 17;29(5):700–18. doi: 10.1177/1049732318809940 (PMC6533803; doi:10.1177/1049732318809940)
Supplement: BBSinCRTS_Table5__Resubmission_Supplementary_20180803 – Supplemental material for Value and Limitations of Broad Brush Surveys Used in Community-Randomized Trials in Southern Africa [file BBSinCRTS_Table5__Resubmission_Supplementary_20180803.pdf]

Table 5: Ethical Clearances

**CRT/Ancillary Study**	**Ethics clearance for BBS**
ZAMSTAR	University of Zambia, Stellenbosch University and the London School of Hygiene and Tropical Medicine ethics committees approved the study in 2004, including BBS. Additional approval obtained for Murray's Master's analysis of BBS in 2007 from Stellenbosch University ethics committee (Murray 2010).
CODA	University of Stellenbosch Health Research Ethics Committee (N04/10/173), the University of Zambia Biomedical Research Ethics Committee (007-10-04), and the London School of Hygiene and Tropical Medicine Ethics Committee (A211 3008) approved the study in 2010, including BBS.
BHOMA	University of Alabama at Birmingham, the University of North Carolina, the University of London School of Hygiene and Tropical Medicine and the University of Zambia Research Ethics Committees approved the study in 2011, including BBS (004-12-08).).
HPTN 071 (PopART)	University of Zambia Humanities and Social Sciences Research Ethics Committee (011-11-12),), Stellenbosch University Health Research Ethics Committee (N12/09/056) and the London School of Hygiene and Tropical Medicine (6278) ethics committees approved BBS ahead of and independent of the main study in 2012.
P-ART-Y	University of Zambia (011-11-12),), Stellenbosch University and the London School of Hygiene and Tropical Medicine ethics committee approved the study in 2015, including the BBS.
Society in Transition	University of KwaZulu-Natal Biomedical Research Ethics Committee (BE197/15) approved the study in 2015.
