# Supplementary material for: Value and Limitations of Broad Brush Surveys Used in Community-Randomized Trials in Southern Africa
Source: Qual Health Res. 2018 Dec 17;29(5):700–18. doi: 10.1177/1049732318809940 (PMC6533803; doi:10.1177/1049732318809940)
Supplement: QHR809940_Supplemental_Material_REV4 – Supplemental material for Value and Limitations of Broad Brush Surveys Used in Community-Randomized Trials in Southern Africa [file QHR809940_Supplemental_Material_REV4.pdf]

Supplemental Table. BBS Participant Types and Numbers and Structured Observations.

CRT	Group Discussions: Local Health Committees			Group Discussions: Stakeholders/Health Experts			Group Discussions: Community Members			In-depth Interviews (IDIs)/ Key Informant Interviews (KIIs)		Structured Observations				
	Groups (*n*)	Participants		Groups (*n*)	Participants		Groups (*n*)	Participant		Participant		Transect Walk	Health Facility	Gathering Places[a]	Exit/Entry Points	Night Walks
		Men	Women		Men	Women		Men	Women	Men	Women					
ZAMSTAR				24								24	24	120	24	24
CODA				16								16	16	112	16	16
BHOMA	16	68	68	16	144	144	62	205	273	34	44	16	16	106	16	16
PopART	16	78	101	36[b]	74	250	78	250	342	44	51	21	39[c]	89	42	12
P-ART-Y	0	0	0	0[d]	0	0	0[e]	0	0	0	0	30	30	0[f]	0	0
Africa Health Research Institute (AHRI)	0	0	0	5	6	30	17	72	83	12	27	4	1	11	2	2

Note. BBS = Broad Brush Survey; CRT = community-randomized trial; ZAMSTAR = Zambia South Africa TB and HIV Reduction; TB = tuberculosis; CODA = Contact Observations of Daily Activities; BHOMA = Better Health Outcome through Mentoring and Assessment; P-ART-Y = PopART for Youth.

[a]Gathering places included bars, funerals, mortuaries, graveyards, gathering places for younger and older men, gathering places younger and older women, hair salons, barbers, watering points, markets, churches, and pension pay-points.

[b]Includes participants identified as "HIV specialists" and "People living with HIV."

cIncludes ART health facilities, antenatal care, and Medical Male Circumcision (MMC) services.

[d]Stakeholders were visited individually, and informal unstructured conversations were conducted.

[e]Informal conversations were conducted with numerous community members across South Africa and Zambia. However, exact numbers were not recorded.

[f]A number of informal observations were conducted during the transect walks.
